# A sensitive and rapid method of lead detection using nanoparticle technology based on monoclonal antibody

**DOI:** 10.3389/fbioe.2022.962230

**Published:** 2022-09-20

**Authors:** Kunzhi Jia, Ming Lin, Qiang Zhao, Mingke Dong, Sumei Ling, Shihua Wang

**Affiliations:** State Key Laboratory of Ecological Pest Control for Fujian and Taiwan Crops, Key Laboratory of Pathogenic Fungi and Mycotoxins of Fujian Province, School of Life Sciences, Fujian Agriculture and Forestry University, Fuzhou, China

**Keywords:** lead (Pb), monoclonal antibody, AuNF-based strip, sensitivity, rapid detection

## Abstract

Lead (Pb) threatens public health due to its toxicity and nonbiodegradable characteristics. It is of significance to develop a sensitive and rapid method for Pb detection. In this study, monoclonal antibodies against Pb were screened with a high affinity constant (K_aff_) of 3.56 × 10^9^ L/mol. Au nanosphere particles (AuNS) and Au nanoflower particles (AuNF) were synthesized with a diameter of 15 nm and 60 nm, respectively. The specific anti-Pb antibodies were then immobilized on AuNS and AuNF for probe development. At last, AuNS- and AuNF-based strips were successfully assembled for comparative study, which were able to effectively detect environmental Pb in 10 min. The limits of detection (LODs) were determined to be 3.91 ng/ml and 0.2 ng/ml, respectively. Thus the developed method provides a feasible solution for sensitive and rapid detection of Pb on site, which is beneficial to food safety and pollution control.

## 1 Introduction

Heavy metal contamination in the environment has significantly increased during the last decade because of electronic waste, mining, smelting, and so on (Sarma et al., 2019). Even traces of nonessential heavy metals (As, Hg, Pb, etc.) are lethal to living animals due to their nonbiodegradable characteristics (Ge et al., 2012). Among these heavy metals, lead (Pb) is typical and widespread. Intracellular Pb usually induces oxidative stress by generation of free radicals and reduction of the antioxidant property of the cell, causing damage to DNA, proteins, and lipids (Ercal et al., 2001; Wu et al., 2016). This cellular toxicity tends to permanently harm the brain, immune system, and kidneys, affecting people’s behavior and growth (Gupta et al., 2012; Dorea, 2019). It is worth noting that people are exposed to high risk of environmental Pb exposure due to increasing Pb contamination. Considering the harm of Pb, the potential exposure to Pb is worthy of attention. Therefore, it is beneficial and meaningful to ensure people’s health by developing a rapid and sensitive method for Pb detection in food or environmental samples.

Conventional analytical methods for Pb are atomic absorption spectroscopy (AAS), inductively coupled plasma mass spectrometry (ICP-MS), and so on, which are considered sensitive and of high accuracy (Xie et al., 2008; Rui & Hao, 2012). However, these methods are usually based on large and expensive laboratory equipment with professional procedures, which are inconvenient for general public to use at home. In contrast, the immunological assay is widely applied for its high sensitivity and specificity. Zhu et al. (2007) developed an antibody to detect Pb concentration in water. Kuang et al.(2013) reported that the visual limit of detection (LOD) can reach 2 ng/ml of Pb using the immunological method. With the improvement of environmental quality, it is necessary to explore a more sensitive method for rapid detection of Pb, which is meaningful for minimizing the public risk of Pb exposure.

Lateral flow immunoassay (LFIA) has been widely used in food and environmental detection, which works with the migration of a liquid sample through a porous membrane by capillary action (Zhou et al., 2019; Chen et al., 2022). Biological interactions take place at specific detection zones of the membrane to produce a signal that can be visualized through use of colored nanoparticles (Connolly & O', 2017). Therefore, LFIA is suitable for sensitive and rapid detection of environmental samples. In this study, anti-Pb monoclonal antibodies with high affinity were screened out. Then, Au nanosphere (AuNS) and nanoflower particles (AuNF) were developed to immobilize the specific Pb antibodies. At last, AuNS- and AuNF-based strips were assembled and applied for rapid detection of Pb in various tested samples.

## 2 Materials and methods

### 2.1 Materials

Standard samples were purchased from the National Nonferrous Metal and Electronic Analysis Center in China (Beijing, China). Goat anti-mouse IgG antibodies were obtained from Shanghai Yisheng Biotechnology Company (Shanghai, China). Chloroauric acid (HAuCl_4_) was purchased from Shanghai Chemical Reagents (Shanghai, China). SP2/0 myeloma cells were stored in our laboratory. Hypoxanthine, aminopterin, and thymidine supplement (HAT), hypoxanthine and thymidine supplement (HT), and polyethylene glycol 1450 solutions (PEG 1450) were purchased from Sigma-Aldrich (Shanghai, China). Balb/c mice were obtained from Wushi Animal Laboratory (Shanghai, China).

### 2.2 Antigen preparation

Isothiocyanobenzy-EDTA (ITCBE) was dissolved in dimethyl sulfoxide (DMSO) at a concentration of 10 mg/ml. 200 μl ITCBE and 1 ml keyhole limpet hemocyanin (KLH, 10 mg/ml) were slowly added into the HEPES buffer solution (0.01 mol/L, pH 9.0). The reaction mixture (pH 8.0) was stirred at 25°C overnight, and then 130 μl of Pb (II) solution (1 mg/ml) was slowly added. After stirring for a further 6 h, the reaction mixture was dialyzed in PBS (0.01 mol/L, pH 7.4) for 3 days. Then, the conjugated Pb (Pb-ITCBA-KLH, Pb-KLH) was stored at −20°C for further use. Pb-ITCBA-BSA (Pb-BSA) was generated with the same method as Pb-KLH. The Pb contents in antigens (1 mg/ml) were determined by ICP-MS.

### 2.3 Animal immunization and hybridoma screening

6- to 8-week-old mice were immunized subcutaneously using 50 μg–200 μg Pb-KLH antigen mixed with Freund’s complete adjuvant. After the fifth immunization, the titers of tail blood serum from immunized mice were tested with iELISA ( indirect enzyme-linked immunosorbent assay) (Zhang et al., 2018). The titer of the serum was defined as the maximum dilution ratio of the serum, which was able to effectively detect Pb-BSA. The mice with a high titer were further injected intraperitoneally (i.p.) with 100 μg Pb-KLH. Three days later, splenocytes were collected and fused with SP2/0 cells at a ratio of 10:1 with PEG1450 (Zhang et al., 2018; Jia et al., 2019). The fused cells were seeded into 96-well microplates and cultured in RPMI 1640 with a 20% FBS/HAT medium (Zhang et al., 2018). Then, positive hybridoma cells secreting antibodies against Pb were determined by iELISA (Ling et al., 2014; Zhang et al., 2018).

### 2.4 Characterization of the positive hybridoma cells secreting antibodies against Pb

The isotype of mAb was determined with an antibody isotyping kit (Sigma, Shanghai, China). Chromosome analysis was carried out with the Giemsa stain solution as described previously (Yuan et al., 2012; Ling et al., 2014).

### 2.5 Generation and purification of anti-Pb mAb

Antibody generation and purification are performed as described previously (Zhang et al., 2018). In brief, approximately 1 × 10^6^ positive hybridoma cells were injected into the mouse abdominal cavity. After 1 week, the ascites fluid was collected for antibody purification. The anti-Pb mAb was purified with Protein G and analyzed with 10% SDS-PAGE.

### 2.6 Determination of antibody affinity and specificity

The affinity of antibodies against Pb was determined with iELISA as described previously (Zhang et al., 2018). Various concentrations of Pb-BSA (5 μg/ml, 2.5 μg/ml, 1.25 μg/ml, and 0.625 μg/ml) were used as coating antigens, and the affinity constant (K_aff_) was calculated with the published method (Ling et al., 2015). In brief, the antibody concentration ([Ab]) was analyzed at the IC_50_ of various concentrations of Pb-BSA according to the affinity curve using Origin 8.0. The K_aff_ was calculated using the equation (K_aff_ = 0.5*(*n*-1)/(*n* [Ab]_t_-[Ab]). In the equation, [Ab]_t_ and [Ab] represent the antibody concentration at the IC_50_ of the antigen concentration [Ag]_t_ and [Ag], respectively, while n represents the ratio of [Ag]–[Ag]_t_. The specificity of mAb against Pb was determined by icELISA (indirect competitive enzyme-linked immunosorbent assay) (Ling et al., 2014). The limit of detection (LOD) represents the concentration of free antigens that inhibit the 10% binding between antibodies and immobilized antigens, which is calculated using the curve equation with y (B/B0) equal to 0.9. Different ions of metals (Pb, Mg, Zn, Cu, Ca, Cd, Mn, Fe, Cr, and EDTA) were used as competition antigens in this study. The other steps are consistent with the standard protocol of ELISA.

### 2.7 Development of Au nanosphere particles and Au nanoflower particles

AuNS was developed using the citrate reduction method described previously (Ling et al., 2015). The development of AuNF refers to the seeding growth method (Ji et al., 2015). In summary, AuNS was used as gold seeds, and the mixture composed of sodium citrate, hydroquinone, and HAuCl_4_ solution was the growth solution. 750 μl HAuCl_4_ solution (1%), 500 μl AuNS solution, 300 μl sodium citrate, and 1 ml hydroquinone (30 mmol/L) were sequentially and slowly added into 0.1 L deionized water with constant stirring. After overnight reaction, the color of the solution becomes blue. The obtained blue AuNF solution can be stored at 4°C for use. TEM (transmission electron microscope) was used to characterize AuNS and AuNF particles, including the morphology and average size. ZETASIZER was used to analyze the diameter distribution of AuNP and AuNP-based probes.

### 2.8 Probe preparation

To develop AuNS-based probes, 300 μl mAb (0.8 mg/ml) against Pb was added into 10 ml of AuNS solution with gentle stirring. After blocking with BSA, polyethylene glycol 20000 (PEG 20000) was added to the reaction solution for 30 min at room temperature. The obtained AuNS probes were used in AuNS-based strips after centrifugation and resuspension in PBS. For AuNF-based probes, 360 μL mAb against Pb were added into 10 ml of AuNF solution with gentle stirring.

### 2.9 Assembling of Au nanosphere particle- and Au nanoflower particle-based strip

AuNS- and AuNF-based strips were assembled with a sample pad, conjugate pad, nitrocellulose (NC) membrane, and absorption membrane. After synthesis, probes were sprayed onto the conjugate pad. The Pb-BSA and goat anti-mouse IgG antibodies were immobilized on the NC membrane to form the test line (T line) and control line (C line), respectively. The competition reactions were indicated at the T lines on the strips.

### 2.10 Sample pretreatment and detection

Samples including rice powder, sweet potato powder, wheat meal, and cornmeal were used for Pb detection. Samples were dissolved in water before Pb detection. All samples were filtered with a 0.22-μm filter and mixed with an equal volume of EDTA solution (0.1 mM). The prepared samples were used as competing antigens for icELISA and AuNS- or AuNF-based strip detection.

## 3 Results

### 3.1 Screening of hybridoma cells secreting antibody against Pb

**FIGURE 1:**
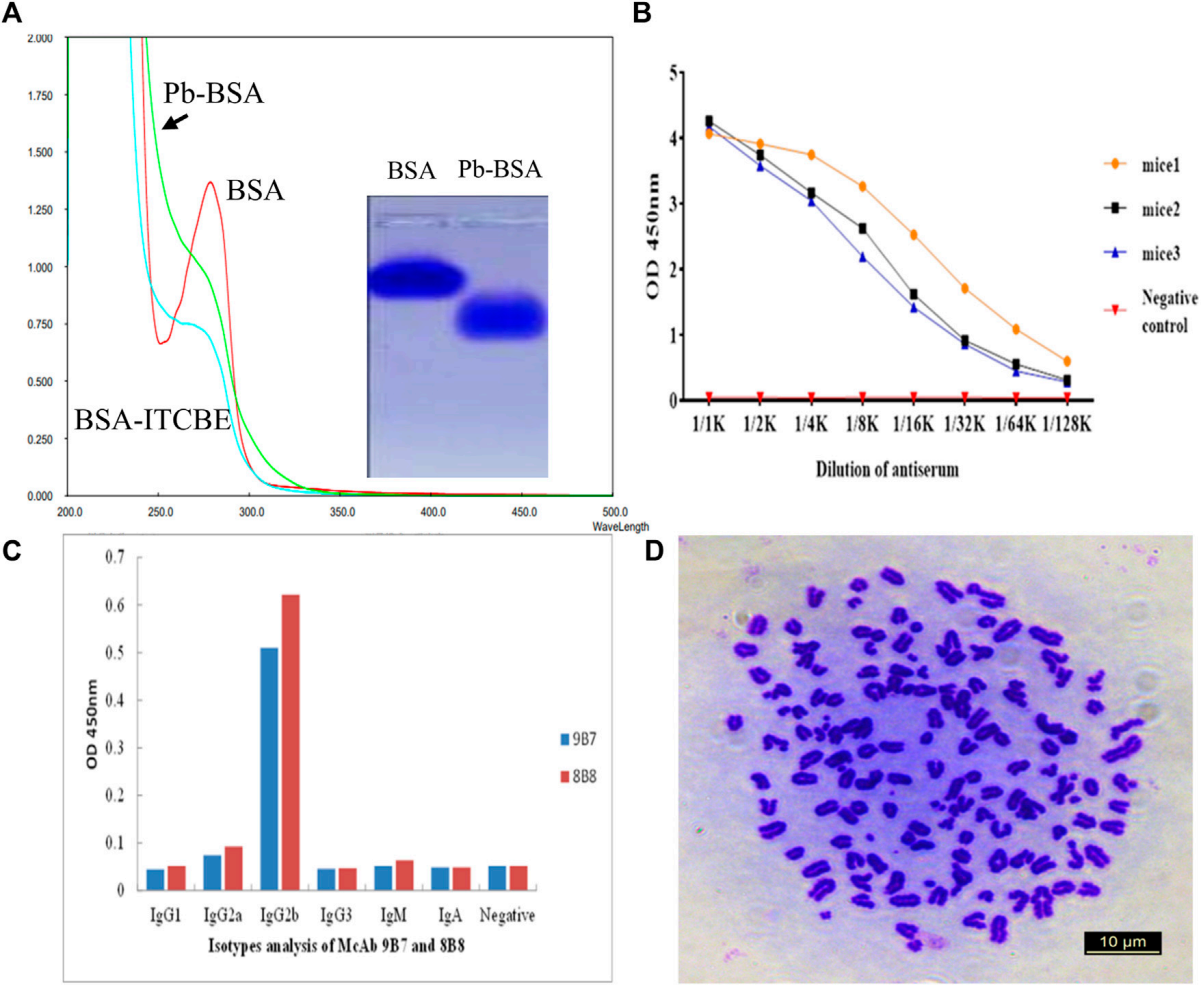
Screening of hybridoma cells secreting antibodies against Pb. **(A)** Analysis of antigens with light absorption scanning and agarose electrophoresis. Pb-BSA: Pb-ITCBE-BSA. **(B)** Titers of mice serum were determined by iELISA. **(C)** Isotype analysis of antibodies secreted by 9B7 and 8B8 cell lines. **(D)** Chromosome analysis of the 9B7 cell line with microscopy.

To generate complete antigen of Pb, a dual function cross-linker ITCBE was used. ITCBE was conjugated with BSA, and Pb was then chelated with ITCBE-BSA, forming Pb-ITCBE-BSA (Pb-BSA). Compared with unconjugated BSA, Pb-BSA exhibits a fast migration velocity and blue shift phenomenon in light absorption peak (Figure 1A), which were consistent with the observations in our previous study (Ling et al., 2021). Pb-ITCBE-KLH (Pb-KLH) was generated with the same method as Pb-BSA. Then, the Pb content in Pb-BSA and Pb-KLH samples was determined by ICP-MS. As shown in Table 1, Pb was not detected in BSA or KLH, while a remarkably high Pb content was detected in Pb-BSA and Pb-KLH. These results indicated that complete antigens for Pb have been successfully generated.

**TABLE 1:** Detection of Pb in complete antigens.

Sample	ICP-MS (μg/L)
BSA	N.D.
Pb-BSA	6,691
KLH	N.D.
Pb-KLH	5,374

N.D., represents not detectable.

After five times of mice immunization with Pb-KLH, mice serum was collected for titer determination with iELISA. Pb-BSA conjugates were used as testing antigens. As shown in Figure 1B, the serum from three immunized mice all exhibited a high titer against Pb. The spleen B cells from the immunized mice were fused with SP/20 cells to produce the hybridoma cell lines. After screening, two cell lines, 9B7 and 8B8, which can secrete anti-Pb antibodies, were successfully obtained. The subtypes of antibodies from 9B7 to 8B8 were both determined as IgG_2b_ (Figure 1C). 9B7, which was found more stable, was chosen for further characterization. As shown in Figure 1D, the chromosome number of the 9B7 cell line was determined as 108 ± 1, approximately equal to the total chromosome number of the spleen cell (39 ± 1) and the SP2/0 myeloma cell (68 ± 2), suggesting that the 9B7 cell line was successfully fused from spleen cells and SP2/0 myeloma cells and qualified for further application.

### 3.2 Generation and characterization of the anti-Pb antibody

**FIGURE 2:**
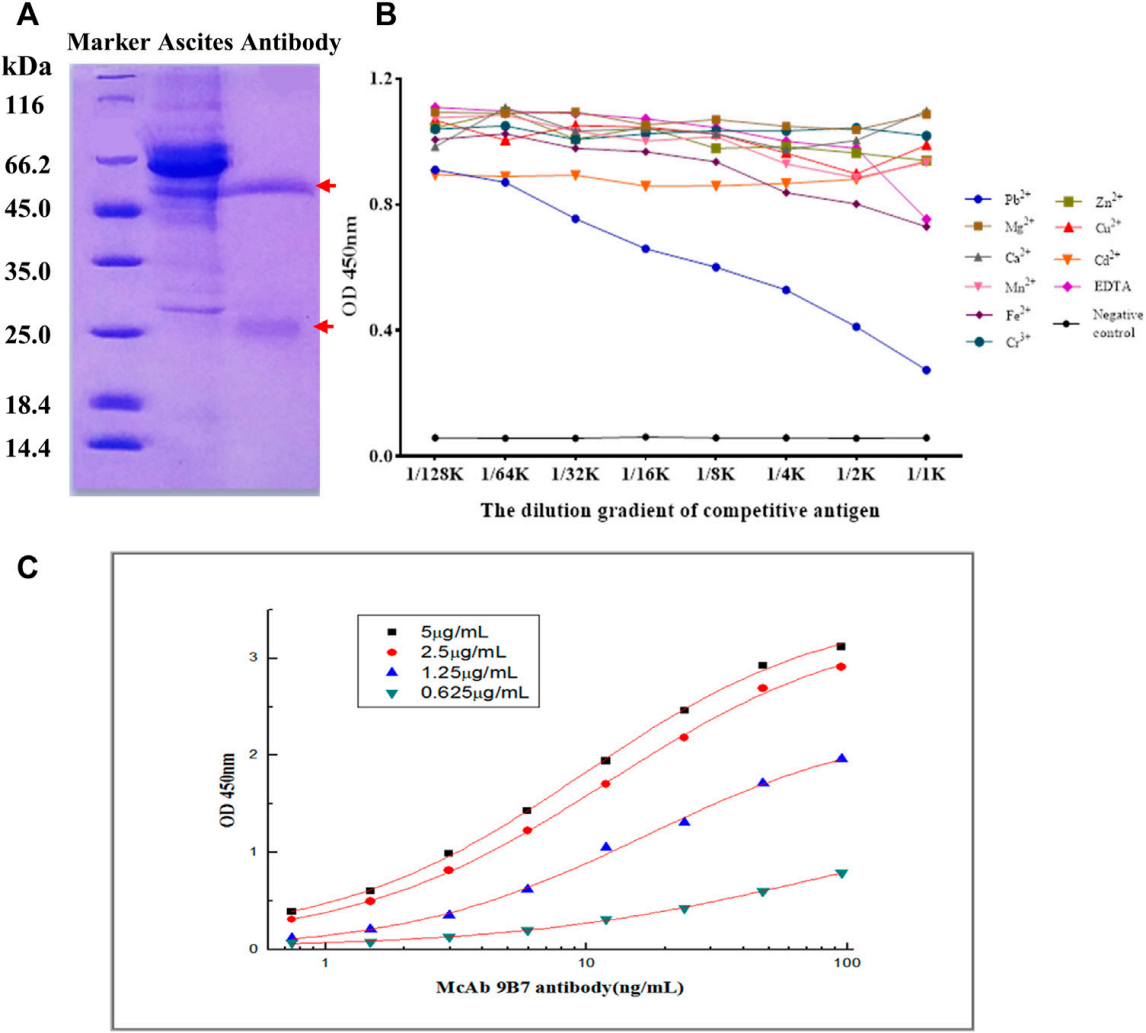
Characterization of 9B7 antibody. **(A)** SDS-PAGE analysis of 9B7 antibodies purified from ascites. **(B)** Specificity of the 9B7 antibody was determined by icELISA. **(C)** Affinity constant of the 9B7 antibody was determined by iELISA.

To gather enough monoclonal antibodies (mAb), 9B7 hybridoma cells were injected into the peritoneal cavity of Balb/c mice. A week later, the mice ascites was collected for purification of anti-Pb mAb. As shown in Figure 2A, two distinct bands, heavy chain and light chain of antibodies, were observed, indicating that 9B7 mAb was successfully purified. The 9B7 antibody was sensitively responsive to Pb but not cross-reactive with other tested metal ions, showing that 9B7 mAb has high specificity to Pb (Figure 2B). The affinity constant of the 9B7 antibody for Pb was measured as K_aff_ = 3.56 × 10^9^ L/mol (Figure 2C), suitable and potentially valuable for further application.

### 3.3 Optimization of experimental conditions for Pb detection by the 9B7 antibody

**FIGURE 3:**
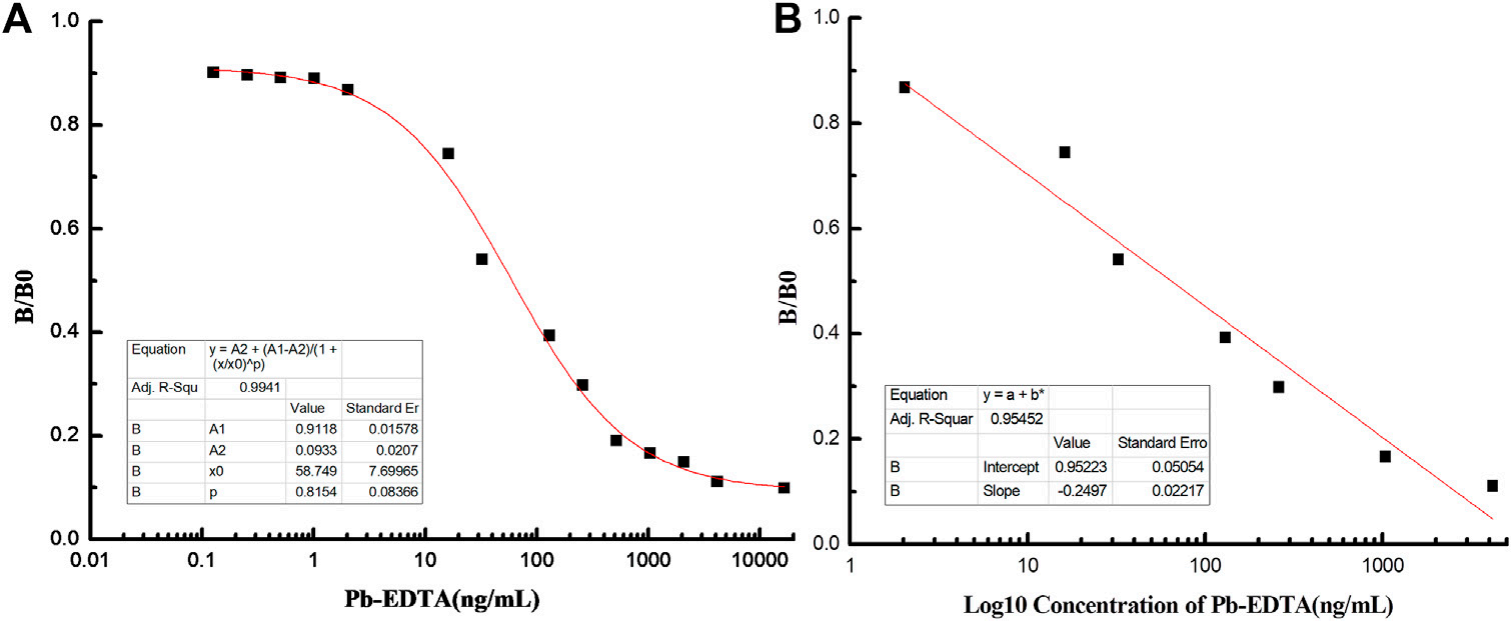
Establishment of the standard curve and linear equation for Pb detection. **(A)** Standard curve of icELISA for Pb detection. **(B)** Linear equation of icELISA for Pb detection.

The absorbance at OD 450 nm was used to measure the reaction of Pb detection. The optimal coating concentration of Pb-BSA was determined to be 5 μg/ml, and the applied concentration of antibody is 22.81 ng/ml. As shown in Figure 3A, B/B0 results were closely correlated with Pb concentrations, and the curve equation was determined to be y = 0.0933 + 0.8185/(1+(x/58.75)ˆ0.8145), with *R* ^2^ = 0.9941. The range of linear Pb detection was 0.0018 μg/ml–2.29 μg/ml (Figure 3B), covering the potential Pb concentration of most environmental samples. The value of IC_50_ was determined to be 58.75 ng/ml, and the limit of detection (LOD) was 0.18 ng/ml. These results showed that the 9B7 antibody has high sensitivity and a good linear range for Pb detection.

### 3.4 Development of gold nanoparticles and AuNP-based probes

**FIGURE 4:**
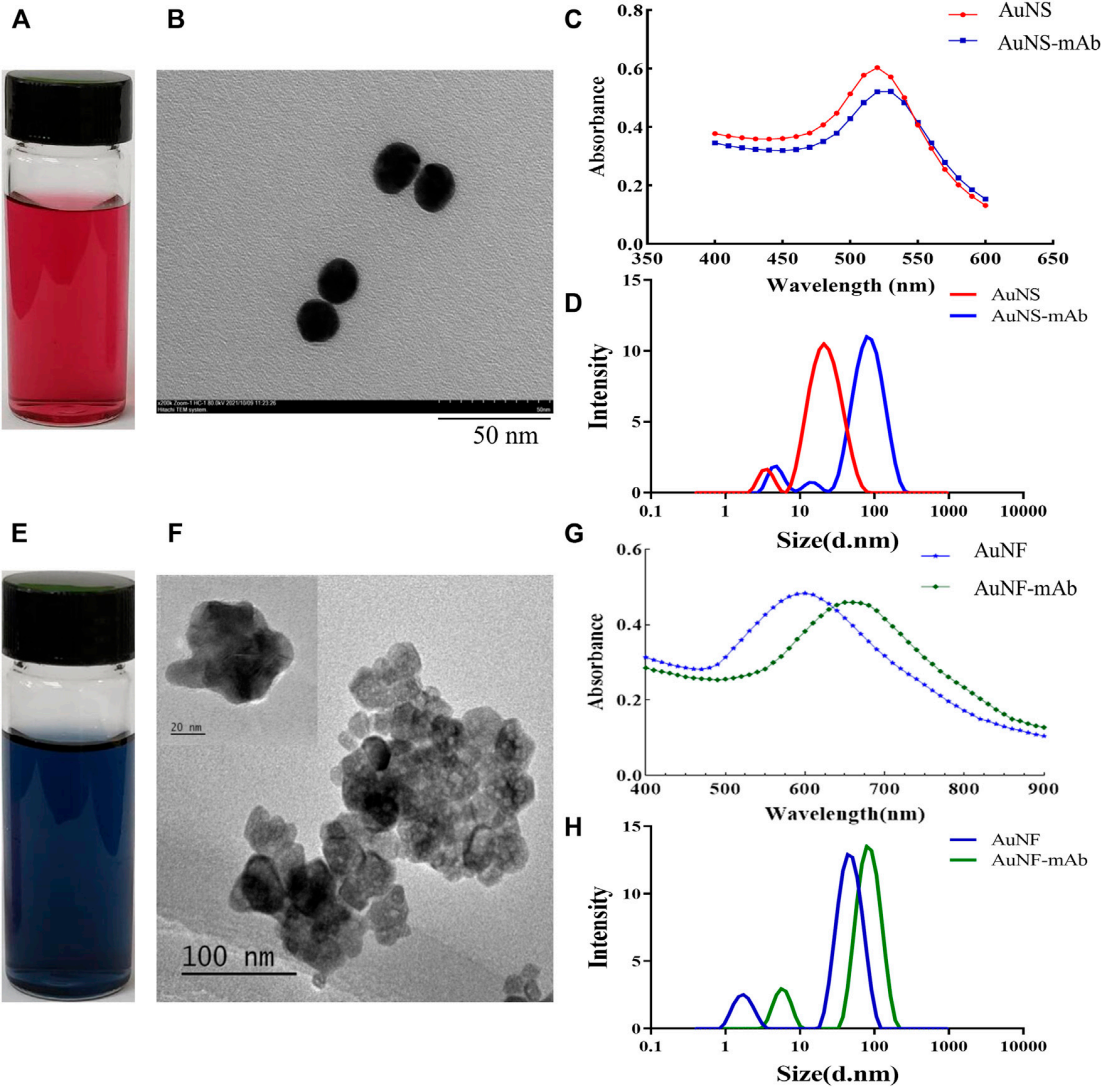
Characterization of Au nanoparticles. **(A,E)** Profile of AuNS and AuNF particles. **(B,F)** Diameters of AuNS and AuNF particles. **(C)** Light absorption of AuNS- and AuNS-labeled 9B7 antibodies. **(D)** Diameter distribution of AuNS and AuNS-labeled 9B7 antibodies. **(G)** Light absorption of AuNF and AuNF-labeled 9B7 antibodies. **(H)** Diameter distribution of AuNF and AuNF-labeled 9B7 antibodies.

Two types of gold nanoparticles (AuNP), Au nanoshpere (AuNS) and Au nanoflower (AuNF), have been developed in this study. The AuNS particles, which exhibit red color (Figure 4A), have a diameter of about 15 nm and maximum absorbance of 520 nm (Figures 4B–D). In contrast, AuNF particles, exhibiting blue color (Figure 4E), have a diameter of about 60 nm (Figure 4F) and maximum absorbance of 600 nm (Figure 4G). AuNPs were then used to label 9B7 antibodies as probes for Pb detection. As shown in Figures 4C,G, compared with AuNS and AuNF, AuNS- and AuNF-labeled antibodies have maximum absorbance at 625 nm and 670 nm, respectively, showing an observable red shift. The phenomenon of the red shift was possibly due to the changed component and diameter with antibodies attaching to AuNP particles, consistent with our previous results (Ling et al., 2021). To further characterize the conjugation of antibodies and AuNPs, the diameters of AuNP particles have also been measured. Compared with AuNPs, the AuNP-based probes exhibited an observable shift of diameters (Figures 4D,H), which was due to the attached antibodies. These phenomena also signaled that antibody labeling was successful. These AuNS- and AuNF particle-labeled 9B7 antibodies were then used as probes for the AuNP-based strips.

### 3.5 Assembling of Au nanosphere particle- and Au nanoflower particle-based strip

**FIGURE 5:**
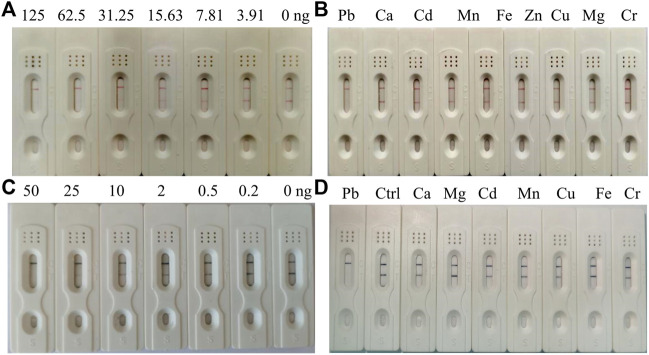
Characterization of the AuNS- and AuNF-based strip test. **(A)** Sensitivity of the AuNS-based strip test. **(B)** Specificity assay of the AuNS-based strip test. **(C)** Sensitivity of the AuNF-based strip test. **(D)** Specificity assay of the AuNF-based strip test.

The AuNP-based strip was composed of a sample pad, conjugate pad, NC membrane, and absorption pad. The sample pad is used for sample loading, and the conjugate pad is sprayed with AuNS- or AuNF-labeled antibodies. In this study, the optimal labeling concentration of antibodies was determined to be 24 μg/ml and 28.8 μg/ml for AuNS and AuNF, respectively. The NC membrane including test and control zone is used for analyte detection. The Pb-BSA coating antigens and the goat anti-mouse IgG antibodies were immobilized in the test zone and control zone, respectively. To evaluate the AuNP-based strip, different concentrations of Pb are used as the analyte. Compared with the control line (C), a weaker color at the test line (T) was defined to be a positive signal. As shown in Figure 5A, 3.91 ng/ml–125 ng/ml of Pb was effectively detected with an AuNS-based strip. In contrast, 0.2 ng/ml–50 ng/ml of Pb has been effectively detected with an AuNF-based strip (Figure 5C). The developed AuNP-based strips were not cross-reactive with other representational ions, including Ca, Cd, Cu, and Cr, at the concentration of 200 ng/ml (Figures 5B,D), indicating that the AuNP-based strip in our study was specific for Pb detection. Compared with the AuNS-based strip, the AuNF-based strip is highly sensitive and relatively suitable for Pb detection in tested samples, which is potentially valuable for Pb detection in real samples.

### 3.6 Application of Au nanoflower particle-based strips in real samples

Considering the detection sensitivity, the AuNF-based strips were further used for Pb detection in real samples, including rice, sweet potato, wheat meal, and cornmeal. As shown in Table 2, Pb has not been detected in rice, sweet potato, and wheat meal, in which the concentration of Pb was less than 0.2 ng/ml determined by ICP-MS. In contrast, Pb was detected in the cornmeal sample, in which the concentration of Pb was determined to be 0.29 ng/ml (Table 2). These results indicate that our AuNF-based strips are effective and suitable for on-site Pb detection in real samples. Moreover, the detection results were displayed in 10 min, showing the valuable prospect for rapid detection of Pb. Also, this strip test is supposed to be potentially effective for other common samples.

**TABLE 2:** Detection of Pb in real samples.

Sample	AuNF-based strip	ICP-MS (μg/L)
Rice	−	< 0.01
Sweet potato	−	0.1519
Wheat meal	−	0.0878
Cornmeal	**+**	0.2895

The symbol “-” represents a negative result, and “+” represents a positive result.

## 4 Discussion

With the increasing in population, it is necessary to develop a rapid method to detect lead (Pb), which threatens the health of human beings. To detect Pb, monoclonal antibody (mAb) has been proven to be effective (Zhu et al., 2007), but the traditional method of ELISA using antibodies is time-consuming. At the same time, it provides an opportunity for fast detection of Pb using a monoclonal antibody. To generate a complete antigen of Pb, a dual-function cross-linker ITCBE was successfully used to conjugate Pb with a carrier protein. To avoid the denaturation of carrier protein, Pb is advised to be added at the last step during the conjugation as described previously. Combined with previous results (Ling et al., 2021), our study suggested that agarose electrophoresis and light absorption were feasible methods to examine the result of carrier proteins and heavy ions (Figures 1A,B). 9B7 monoclonal antibody, exhibiting a high affinity for Pb (Figure 2), has been screened in this study. 9B7 mAb was shown to detect Pb with a good linear range (Figure 3B) using icELISA, which is valuable for Pb detection. Although the ELISA method using the 9B7 antibody was also effective for detecting Pb in samples, more than 2 h were necessary for this method due to the experimental procedure (Ling et al., 2014; Zhang et al., 2018). For example, Zhou et al.(2011) reported an enhanced ELISA method for sensitive detection of Pb, but the method took around 8 h–10 h, including antigen coating. In such a case, it is hardly satisfying for the requirement of rapid detection on site. Considering that LFIA has been widely used in fields of rapid detection (Zhou et al., 2020; Chen et al., 2022), two types of AuNP (AuNS and AuNF), which have different diameters and shapes (Figure 4), have been developed for establishment of LFIA probes in this study. Two types of AuNP-based strips were all effective for Pb detection (Figure 5), which are suitable for rapid Pb detection. Moreover, the AuNF-based strip is around 20-fold more sensitive than the AuNS-based strip for Pb detection in our comparative experiment (Figure 5), which is reasonable (Zhang et al., 2015; Ling et al., 2021) and meaningful. Why the AuNF-based strip is more sensitive than AuNS for Pb detection? In our study, the main differentiating factors are diameter and shape (Figure 4). This bigger diameter and multi-branched flower shape seem to be beneficial to antibody association. In this study, the optimal concentration of antibodies for AuNF labeling is 28.8 μg/ml versus 24 μg/ml for AuNS. On the other hand, AuNF exhibits higher optical brightness, which was possibly due to the tips of AuNF and large-size diameter (Xu et al., 2016; Chen et al., 2022).Thus, more sensitive LFIA methods of Pb detection are theoretically possible through tailoring nanoparticle designs (Zhou et al., 2019; Chen et al., 2022; Duan et al., 2022). Although the detailed mechanism is unclear, the AuNF-based strip is more sensitive for Pb detection in this study. Considering the increasing requirements of environment quality, AuNF-based strip is potentially valuable in the future. The Pb detection in real samples with an AuNF-based strip further proved that the AuNF-based strip test is effective and promising for rapid detection of Pb in various food and environmental samples.

In summary, 9B7 anti-Pb mAb with high affinity has been screened, and then two types of AuNP-based strips were successfully assembled using AuNP probes with 9B7 antibodies. The AuNS- and AuNF-based strips were all effective for rapid detection of Pb ions. Furthermore, our study indicated for the first time that AuNF-based strips showed around 20-fold more sensitivity in Pb detection than AuNS-based strips. AuNF-based strips also effectively detect Pb in real samples, providing a feasible solution for rapid detection of Pb in daily samples, which is beneficial to food safety and public health.

## Data Availability

The original contributions presented in the study are included in the article/Supplementary Material; further inquiries can be directed to the corresponding authors.

## References

[B1] Chen X. Ding L. Huang X. Xiong Y. (2022). Tailoring noble metal nanoparticle designs to enable sensitive lateral flow immunoassay. Theranostics 12 (2), 574–602. 10.7150/thno.67184 34976202 PMC8692915

[B2] Connolly R. O' K. R. (2017). Magnetic lateral flow immunoassay test strip development - considerations for proof of concept evaluation. Methods 116, 132–140. 10.1016/j.ymeth.2017.02.002 28213280

[B3] Dorea J. G. (2019). Environmental exposure to low-level lead (Pb) co-occurring with other neurotoxicants in early life and neurodevelopment of children. Environ. Res. 177, 108641. 10.1016/j.envres.2019.108641 31421445

[B4] Duan H. Ma T. Huang X. Gao B. Zheng L. Chen X. (2022). Avoiding the self-nucleation interference: a pH-regulated gold *in situ* growth strategy to enable ultrasensitive immunochromatographic diagnostics. Theranostics 12 (6), 2801–2810. 10.7150/thno.70092 35401815 PMC8965500

[B5] Ercal N. Gurer-Orhan H. Aykin-Burns N. (2001). Toxic metals and oxidative stress part I: mechanisms involved in metal-induced oxidative damage. Curr. Top. Med. Chem. 1 (6), 529–539. 10.2174/1568026013394831 11895129

[B6] Ge F. Li M. M. Ye H. Zhao B. X. (2012). Effective removal of heavy metal ions Cd2+, Zn2+, Pb2+, Cu2+ from aqueous solution by polymer-modified magnetic nanoparticles. J. Hazard. Mater. 211-212, 366–372. 10.1016/j.jhazmat.2011.12.013 22209322

[B7] Gupta V. K. Ali I. Saleh T. A. Nayak A. Agarwal S. (2012). Chemical treatment technologies for waste-water recycling-an overview. RSC Adv. 2 (16), 6380–6388. 10.1039/c2ra20340e

[B8] Ji Y. Ren M. Li Y. Huang Z. Shu M. Yang H. (2015). Detection of aflatoxin B(1) with immunochromatographic test strips: Enhanced signal sensitivity using gold nanoflowers. Talanta 142, 206–212. 10.1016/j.talanta.2015.04.048 26003713

[B9] Jia K. Zhang D. Wang Y. Liu Y. Kong X. Yang Q. (2019). Generation and characterization of a monoclonal antibody against human BCL6 for immunohistochemical diagnosis. PLoS One 14 (5), e0216470. 10.1371/journal.pone.0216470 31063496 PMC6504089

[B10] Kuang H. Xing C. Hao C. Liu L. Wang L. Xu C. (2013). Rapid and highly sensitive detection of lead ions in drinking water based on a strip immunosensor. Sensors 13 (4), 4214–4224. 10.3390/s130404214 23539028 PMC3673080

[B11] Ling S. Chen Q. A. Zhang Y. Wang R. Jin N. Pang J. (2015). Development of ELISA and colloidal gold immunoassay for tetrodotoxin detetcion based on monoclonal antibody. Biosens. Bioelectron. X. 71, 256–260. 10.1016/j.bios.2015.04.049 25913446

[B12] Ling S. Pang J. Yu J. Wang R. Liu L. Ma Y. (2014). Preparation and identification of monoclonal antibody against fumonisin B(1) and development of detection by Ic-ELISA. Toxicon 80, 64–72. 10.1016/j.toxicon.2013.12.008 24378835

[B13] Ling S. Zhao Q. Iqbal M. N. Dong M. Li X. Lin M. (2021). Development of immunoassay methods based on monoclonal antibody and its application in the determination of cadmium ion. J. Hazard. Mater. 411, 124992. 10.1016/j.jhazmat.2020.124992 33454572

[B14] Rui Y. Hao J. (2012). Determination of nine heavy metals by inductively coupled plasma mass spectroscopy in groundwater from northeast rural of China. Asian J. Chem. 24 (6), 2825–2826.

[B15] Sarma G. K. Sen G. S. Bhattacharyya K. G. (2019). Nanomaterials as versatile adsorbents for heavy metal ions in water: a review. Environ. Sci. Pollut. Res. 26 (7), 6245–6278. 10.1007/s11356-018-04093-y 30623336

[B16] Wu X. Cobbina S. J. Mao G. Xu H. Zhang Z. Yang L. (2016). A review of toxicity and mechanisms of individual and mixtures of heavy metals in the environment. Environ. Sci. Pollut. Res. 23 (9), 8244–8259. 10.1007/s11356-016-6333-x 26965280

[B17] Xie F. Lin X. Wu X. Xie Z. (2008). Solid phase extraction of lead (II), copper (II), cadmium (II) and nickel (II) using gallic acid-modified silica gel prior to determination by flame atomic absorption spectrometry. Talanta 74 (4), 836–843. 10.1016/j.talanta.2007.07.018 18371717

[B18] Xu P. Li J. Huang X. Duan H. Ji Y. Xiong Y. (2016). Effect of the tip length of multi-branched AuNFs on the detection performance of immunochromatographic assays. Anal. Methods 8 (16), 3316–3324. 10.1039/c5ay03274a

[B19] Yuan J. Zhang C. Fang S. Zhuang Z. Ling S. Wang S. (2012). A monoclonal antibody against F1-F0 ATP synthase beta subunit. Hybrid. (Larchmt) 31 (5), 352–357. 10.1089/hyb.2012.0033 23098302

[B20] Zhang D. Xie C. Wang R. Yang Q. Chen H. Ling S. (2018). Effective preparation of a monoclonal antibody against human chromogranin A for immunohistochemical diagnosis. BMC Biotechnol. 18 (1), 25. 10.1186/s12896-018-0436-z 29728076 PMC5935939

[B21] Zhang L. Huang Y. Wang J. Rong Y. Lai W. Zhang J. (2015). Hierarchical flowerlike gold nanoparticles labeled immunochromatography test strip for highly sensitive detection of *Escherichia coli* O157:H7. Langmuir 31 (19), 5537–5544. 10.1021/acs.langmuir.5b00592 25919084

[B22] Zhou S. Xu L. Kuang H. Xiao J. Xu C. (2020). Immunoassays for rapid mycotoxin detection: state of the art. Analyst 145 (22), 7088–7102. 10.1039/d0an01408g 32990695

[B23] Zhou Y. Ding L. Wu Y. Huang X. Lai W. Xiong Y. (2019). Emerging strategies to develop sensitive AuNP-based ICTS nanosensors. TrAC Trends Anal. Chem. 112, 147–160. 10.1016/j.trac.2019.01.006

[B24] Zhou Y. Tian X. L. Li Y. S. Pan F. G. Zhang Y. Y. Zhang J. H. (2011). An enhanced ELISA based on modified colloidal gold nanoparticles for the detection of Pb(II). Biosens. Bioelectron. X. 26 (8), 3700–3704. 10.1016/j.bios.2011.02.008 21371875

[B25] Zhu X. Hu B. Lou Y. Xu L. Yang F. Yu H. (2007). Characterization of monoclonal antibodies for lead-chelate complexes: applications in antibody-based assays. J. Agric. Food Chem. 55 (13), 4993–4998. 10.1021/jf070787d 17547420

